# Maladaptive Emotion Regulation Among Empty-Nest Older Adults with Depressive Symptoms Across Interpersonal Contexts

**DOI:** 10.3390/bs16020263

**Published:** 2026-02-11

**Authors:** Junni Wang, Yu Chai, Zhao Yao

**Affiliations:** 1 School of Marxism, Xi’an University of Science and Technology, Xi’an 710600, China; wjunni@xust.edu.cn; 2 School of Foreign Studies, Xi’an Jiaotong University, Xi’an 710049, China; chaiyu@stu.xjtu.edu.cn

**Keywords:** empty-nest older adults, depressive symptoms, interpersonal contexts, emotion regulation, strategy flexibility

## Abstract

Depressive symptoms are common among empty-nest older adults in China, yet the interpersonal emotion regulation patterns linked to these symptoms remain unclear. We compared interpersonal emotion regulation strategies in sadness and anger contexts between empty-nest older adults with high and low depressive symptoms (N = 129). Participants reported passive, proactive, and problem-solving strategies, and cross-context variability was used to index regulatory flexibility. Results showed that the high depressive symptom group used fewer passive strategies (e.g., acceptance, avoidance/denial) across both contexts and showed lower cross-context variability. In sadness, they employed more suppression but fewer Express and Solve strategies (e.g., communication, advice-seeking, planning, and problem-solving); in anger, they used more Express and Seek strategies (e.g., expressing and understanding feelings). These findings suggest that depressive symptoms in empty-nest older adults correlate with a maladaptive regulatory style, marked by reduced passive engagement, less proactive involvement, and more suppression in sadness, more inward aggression in anger, and limited cross-context flexibility.

## 1. Introduction

In China, the population of empty-nest older adults—those aged 60 and above living alone or only with a spouse—is substantial. According to data from the Seventh National Population Census, the number of empty-nest older adults reached 147 million in 2020, accounting for 55.5% of the elderly population, and this proportion is projected to rise to 90% by 2030 (D. Su et al., 2012). A key concern within this population is depression. For example, a systematic meta-analysis indicates that the prevalence of depression among empty-nest older adults is as high as 38.6% (Zhang et al., 2020). However, the psychological impact of the empty-nest situation is heterogeneous (Hartanto et al., 2024; Feng & Phillips, 2024; S. Xu et al., 2023)—that is, while some older adults remain psychologically healthy in the same empty-nest circumstances, others develop depression. Changes during the empty-nest phase (e.g., role transitions and social adjustments) pose challenges for adapting to this new stage. Within the collectivist cultural context of China, cultivating broader patterns of social engagement promotes well-being (J. Su et al., 2022; Grünjes et al., 2024; Kim & Jung, 2022), whereas effective interpersonal emotion regulation is crucial for preventing depression (Williams et al., 2018; Messina et al., 2023; Dixon-Gordon et al., 2015). Research also shows that depressed individuals often exhibit maladaptive interpersonal behaviors, which are associated with impaired ability to regulate emotions (Fearey et al., 2021). From an emotion regulation perspective, this study examines how empty-nest older adults regulate emotions in interpersonal contexts. By contrasting high versus low depressive symptom groups, we identified strategy profiles associated with depressive symptoms.

Previous research has often examined differences in the use of emotion regulation strategies in two common interpersonal contexts: sadness and anger (Coats & Blanchard-Fields, 2008; Etxeberria et al., 2016). These emotion regulation strategies are typically classified into three categories: passive, proactive (i.e., Express and Seek), and problem-solving (Coats & Blanchard-Fields, 2008; Etxeberria et al., 2016). Passive strategies involve avoiding conflict without overt engagement, such as suppression, avoidance, acceptance, or denial (Etxeberria et al., 2016; Daros et al., 2021). Proactive strategies refer to confronting negative emotions in order to manage them and include Express (e.g., venting emotions and communicating with the person involved or with other friends) and Seek (e.g., seeking emotional support, searching for information, or attributing the cause of emotions) (Blanchard-Fields & Coats, 2008; Blanchard-Fields et al., 2004). Problem-solving strategies—often described as the most adaptive—entail deliberate attempts to directly address or resolve the conflict (Aldao et al., 2010; Al-Refae et al., 2021).

The use of passive strategies by empty-nest older adults may be moderated by interpersonal contexts. Individuals with low depressive symptoms often maintain emotional balance by adapting to role changes in the empty-nest phase, which involves selecting context-appropriate regulatory strategies—including the judicious use of passive approaches (Blanke et al., 2020; Blanchard-Fields & Coats, 2008). Conversely, following this logic, older adults with high depressive symptoms would be expected to struggle with flexibly applying passive strategies in accordance with situational demands. Furthermore, depressed and healthy individuals exhibit inconsistent use of passive-strategy subtypes. The former rely more on avoidance in interpersonal conflicts (Thompson et al., 2018; Constantino et al., 2008) and yet avoid less in anger contexts (C. Xu et al., 2022), frequently use suppression, and seldom employ acceptance (Nezlek & Kuppens, 2008; Joormann & Stanton, 2016). In contrast, healthy individuals are more likely to use acceptance and effectively avoid interpersonal conflicts (Kökönyei et al., 2024; I. Mueller et al., 2024; Blanchard-Fields & Coats, 2008). This suggests that the use of passive strategies and their subtypes by empty-nest older adults may differ depending on the level of depressive symptoms.

Regarding the use of proactive and problem-solving strategies, differences may also exist between empty-nest older adults with high and low depressive symptoms across different contexts. Given that the use of proactive strategies is indicative of better psychological health (Daros et al., 2021), empty-nest older adults with low depressive symptoms are able to effectively employ such proactive approaches. Compared to sadness, anger tends to provoke stronger emotional arousal and causes more harm to interpersonal harmony. Therefore, the emotion regulation strategies involved in these two contexts differ (Blanchard-Fields, 2007; Blanchard-Fields et al., 2004; C. Xu et al., 2022). Empty-nest older adults with low depressive symptoms are likely to recognize different emotional situations and respond flexibly—using proactive strategies when appropriate, and avoiding them when not—to maintain emotional stability and good relationships (Blanchard-Fields et al., 2004; Blanchard-Fields, 2007; Blanchard-Fields & Coats, 2008). For example, they may actively communicate and offer support in sadness-related contexts, while avoiding proactive strategies such as direct confrontation in anger-related situations.

In contrast, empty-nest older adults with high depressive symptoms may show more complex patterns. Depression can impair older adults’ ability to employ proactive emotion regulation strategies (Jayamaha et al., 2021). Additionally, depression is negatively correlated with the use of problem-solving strategies (Aldao et al., 2010). Consequently, empty-nest older adults with high depressive symptoms may use fewer proactive or problem-solving strategies than their counterparts with low depressive symptoms in sadness-related contexts. However, in anger-related interpersonal contexts, depressed individuals often express hostility in covert ways—such as responding to interpersonal threats with compliance rather than direct confrontation (Dawood et al., 2013; Simon et al., 2015; Cain et al., 2012). Therefore, in managing interpersonal anger, empty-nest older adults with high depressive symptoms may engage in covert regulatory strategies as a way of expressing hostility, rather than displaying it overtly. Additionally, while some studies have found that depressed individuals rarely express emotions (Liu et al., 2024), others indicate that seeking comfort and emotional expression can serve as predictors of depression (Messina et al., 2023). This inconsistency suggests that empty-nest older adults with high versus low depressive symptoms may differ in their use of proactive and problem-solving strategies, including their subtypes, across different contexts.

In addition to the link between the use of specific strategies and depressive symptoms in particular contexts, studies have also shown that an individual’s ability to flexibly apply different regulation strategies across situations is also a key factor influencing psychological health (Cheng et al., 2014; Kato, 2012). Existing research has indicated that variability in emotion regulation strategy use across different contexts is negatively associated with depressive symptoms (Wang et al., 2021; Hu & Tamir, 2025). However, the applicability of this finding to empty-nest older adults across different interpersonal contexts—such as sadness and anger—remains to be examined.

In sum, the existing literature indicates that depressed individuals make less use of passive/acceptance coping strategies; they show elevated suppression, reduced anger avoidance, diminished proactivity in managing distress (though findings on expression are inconsistent), more covert aggression when angry, and lower overall flexibility. However, it has yet to be determined whether these patterns manifest in distinct profiles across sadness and anger contexts in empty-nest older adults at different levels of depressive symptoms.

To address this gap, the present study investigates how empty-nest older adults with high versus low depressive symptoms differ in their use of passive, proactive, and problem-solving strategies—as well as their subtypes—across two distinct interpersonal contexts: sadness and anger. It also explores whether and how the two groups differ in their flexibility to shift strategies between these contexts. We hypothesized that: (1) empty-nest older adults with high depressive symptoms may use fewer passive strategies overall, including less acceptance across two contexts, while showing distinct subtype patterns: increased suppression in sadness and decreased avoidance in anger; (2) they would employ fewer proactive and problem-solving strategies in sad contexts; (3) in anger, they may employ certain covert proactive strategies to express their anger; and (4) they would display less variability in strategies across sadness and anger contexts compared with low depressive symptom group.

## 2. Methods

### 2.1. Participants

Adopting a sample size consistent with prior research (Etxeberria et al., 2016; Coats & Blanchard-Fields, 2008), we recruited 202 eligible empty-nest older adults via convenience sampling from a major community of this population in a western Chinese city. All participants were long-term Chinese residents and met the specified criteria for age and living arrangements. The sample comprised a lower–middle-income group, and no participant was under professional psychological/psychiatric care at the time of data collection—a fact confirmed through self-report and family verification. Based on established cut-off scores (participants with a Center for Epidemiologic Studies Depression Scale (CES-D10) (see Supplementary Materials) score of ≤6 were classified as being in the low depressive symptom group, and those with a CES-D10 score of ≥10 were classified as being in the high depressive symptom group) and predefined inclusion criteria, nine participants were excluded (five lost interest and withdrew, two had a history of brain disease with limited mobility, and two did not meet the empty-nest criteria), and sixty-four fell within the middle range of depressive symptom scores (7–9 points) and did not participate in the subsequent study. A total of 129 participants were identified as eligible and were enrolled in the study (Q. B. Huang et al., 2015; G. Huang et al., 2020). Of these, 74 were classified as the low depressive symptom group and 55 as the high depressive symptom group among empty-nest older adults. There was no significant age difference between the two groups (*M*_low depressive symptoms_ = 72.93, *SD* = 6.38; *M*_high depressive symptoms_ = 74.76, *SD* = 6.52; *t*(127) = –1.60, *p* = 0.11). There were also no statistically significant differences between the two groups in terms of gender, education level, economic income, number of diseases, and disease stability (*ps* > 0.05). In addition, while government and community support provided to both groups of empty-nest older adults was identical, individuals with high depressive symptoms (1.13 ± 0.70) interacted significantly less frequently with relatives and friends (0 = almost none, 1 = occasional, and 2 = frequent) than their counterparts with low depressive symptoms (1.88 ± 0.37); *t*(127) = 7.923 and *p* < 0.001.

Standardized screening tools were also used to confirm eligibility: the Community Screening Instrument for Dementia (CSI-D; Hall et al., 2000) excluded cognitive impairment; the 10-item Geriatric Anxiety Scale—Short Form (GAS-10; A. E. Mueller et al., 2015) assessed anxiety symptoms; and the Barthel Index (Mahoney & Barthel, 1965) together with the Lawton Instrumental Activities of Daily Living Scale (Lawton & Brody, 1969) measured functional status.

### 2.2. Procedure

Community staff first conducted a preliminary screening of eligible empty-nest older adults based on the study’s inclusion criteria (aged ≥ 60, living alone/with spouse for ≥1 year, and adequate communication abilities) and obtained their initial consent to participate. Guided by staff, the research team conducted home visits, explained the study’s purpose, emphasized its voluntary and confidential nature, and obtained written informed consent. Each participant received household goods valued at 30 RMB upon survey completion. After the survey, family members of older adults with high depressive symptoms were informed of associated risks and available intervention resources.

Following screening with the CES-D10 and eligibility confirmation, participants filled out a demographic questionnaire. They then read two interpersonal vignettes designed to induce sadness and anger, respectively, and after each scenario, completed the Strategy Questionnaire to report their emotion regulation strategies.

In this study, a paper-and-pencil test was used. Each story and its corresponding questionnaire were printed separately on an A4 sheet, labeled Vignette A (sadness) and Vignette B (anger). The order of story presentation was counterbalanced across participants; half received Story A first, and the other half received Story B first.

### 2.3. Measure

Emotion regulation strategies were assessed using the Strategy Questionnaire developed by Coats and Blanchard-Fields (2008). The original version employed eight vignettes (four sadness, four anger), each followed by 22 items. Following Etxeberria et al. (2016) and considering the cognitive load limitations of older adults, one sadness vignette and one anger vignette were selected and culturally adapted to Chinese norms:

Sadness vignette: “*A close friend of yours has been diagnosed with a serious illness and is undergoing treatment. Upon learning about this situation, you feel sad*.”

Anger vignette: “*A close friend whom you deeply trust falsely accuses you of doing something bad that you did not do. You feel angry about this*.”

A pilot test with 36 older adults (18 with low and 18 with high depressive symptoms), not included in the main study, rated vignette relevance on a 5-point scale (1 = not at all to 5 = very much). Both groups reported moderate identification (*M*_low depressive symptoms_ = 2.97, *SD* = 0.88; *M*_high depressive symptoms_ = 3.08, *SD* = 0.94; *ps* > 0.65), supporting vignette suitability.

Following each vignette, participants rated the likelihood of employing each of the 22 items on a 4-point scale (1 = definitely would not to 4 = definitely would). For example, “I would try to hide my feelings” would receive a score of 4 from someone certain to suppress sadness.

The questionnaire includes four categories of strategies: (1) Passive—avoidance, denial, acceptance, or suppression of emotion; (2) Express—confronting the stressor and venting emotions (e.g., yelling); (3) Seek—pursuing emotional support or informational resources from oneself or others; and (4) Solve—focusing on resolving the problem rather than managing emotion. As conceptualized by (Blanchard-Fields & Coats, 2008; Blanchard-Fields et al., 2004), Express and Seek are viewed as proactive strategies, while Solve reflects direct problem-solving. In this study, internal consistency was acceptable to excellent for sadness: passive strategies (α = 0.68), expressing emotions (α = 0.75), seeking advice (α = 0.81), and problem-solving (α = 0.93); and it was strong for anger: passive strategies (α = 0.75), expressing emotions (α = 0.86), seeking advice (α = 0.86), and problem-solving (α = 0.90).

## 3. Results

Independent-samples *t* tests were conducted to examine differences in emotion regulation strategies between empty-nest older adults with high and low depressive symptoms across interpersonal contexts (sadness vs. anger). Table 1 presents the mean total scores for each strategy in the two groups, along with the *t* test results.

When confronted with interpersonal sadness, empty-nest older adults with high depressive symptoms reported significantly lower use of passive strategies (*p* < 0.05), proactive Express (*p* < 0.05), and Solve strategies (*p* < 0.001) compared with their counterparts with low depressive symptoms. These findings suggest that, in sadness-eliciting contexts, older adults with high depressive symptoms are less inclined to employ passive coping, express emotions proactively, or adopt problem-solving approaches.

In anger-related situations, older adults with high depressive symptoms also used passive strategies significantly less often (*p* < 0.001) than older adults with low depressive symptoms. In contrast, they reported significantly greater use of proactive Express (*p* < 0.05) and Seek (*p* < 0.05). This suggests that, although passive coping is less common among individuals with high depressive symptoms when experiencing anger, they tend to rely more on proactive Express and Seek.

To further clarify which subtypes within these broader strategies were associated with depressive symptoms, additional analyses compared the use of specific subtypes between the two groups (see Table 2).

In sadness-eliciting contexts, empty-nest older adults with high depressive symptoms reported significantly less use of avoidance/denial and acceptance strategies (*ps* ≤ 0.001), but significantly greater use of suppression (*p* < 0.05) compared with their counterparts with low depressive symptoms. For proactive strategies, they reported significantly lower use of communication (*p* < 0.001) and advice-seeking (*p* < 0.05). Within the Solve category, both problem-solving and planning were used significantly less often by older adults with high depressive symptoms (*ps* ≤ 0.001). These findings indicate that, when handling sadness, individuals with high depressive symptoms are less likely to adopt avoidance/denial, acceptance, communication, advice-seeking, and problem-focused strategies, but are more inclined to use suppression.

In anger-eliciting contexts, a similar pattern emerged for passive strategies: older adults with high depressive symptoms also used avoidance/denial and acceptance significantly less often than older adults with low depressive symptoms (*ps* ≤ 0.001). However, they reported significantly greater reliance on emotional expression (*p* < 0.05) and understanding feelings (*p* < 0.001).

In sum, these results suggest that empty-nest older adults with low depressive symptoms tend to rely on avoidance/denial and acceptance when managing both interpersonal sadness and anger. In the context of sadness, they are also more likely to employ proactive communication, advice-seeking, problem-solving, and planning. But in anger situations, they do not employ any proactive or problem-solving strategies. By contrast, older adults with high depressive symptoms only use suppression in sadness, and in anger-related situations, they rely more on emotional expression and reflective processing of anger.

To further examine how strategy use varied by emotional context within each group, paired-samples *t* tests were conducted to compare responses to sadness versus anger scenarios (see Table 3).

Among empty-nest older adults with low depressive symptoms, no significant difference was found in the use of passive strategies across sadness- and anger-eliciting contexts. However, all proactive and problem-solving subtypes differed significantly by these contexts (*ps* < 0.001). Specifically, these participants reported significantly more frequent use of emotional expression, emotional communication, understanding feelings, advice-seeking, support-seeking, problem-solving, and planning when responding to sadness compared to anger. These findings suggest that older adults with low depressive symptoms are more likely to engage in proactive and problem-focused coping in sadness-evoking situations, whereas such patterns are used less frequently in anger-related contexts.

In contrast, among older adults with high depressive symptoms, only two subtypes—emotional suppression and emotional expression—were used significantly more often in response to sadness than anger (*ps* < 0.001). No significant differences were found for the remaining subtypes across the two emotional contexts. This pattern indicates a more constrained and rigid use of coping strategies among older adults with high depressive symptoms, regardless of emotional context.

## 4. Discussion

This study is the first to examine differences in emotion regulation strategies between empty-nest older adults with high and low depressive symptoms across different interpersonal contexts. Results showed that empty-nest older adults with high depressive symptoms used fewer passive strategies (e.g., acceptance, avoidance/denial) across both sadness and anger contexts. In sadness, they employed suppression more often but used fewer Express and Solve strategies (including communication, advice-seeking, planning, and problem-solving). In anger, they utilized more Express and Seek strategies (such as expressing and understanding feelings). This group also showed lower strategy variability across interpersonal situations. The findings contribute to a better understanding of the factors associated with depressive symptoms in the empty-nest population.

### 4.1. Passive Strategies in Interpersonal Contexts

For passive strategies, empty-nest older adults with high depressive symptoms used significantly fewer passive strategies than their counterparts with low depressive symptoms. They also employed suppression more frequently in response to sadness and used both acceptance and avoidance/denial less often across situations. Except for the lower use of avoidance/denial in sadness, these results are consistent with the hypothesis. Prior research suggests that the appropriate use of passive strategies serves a protective function for older adults’ mental health (Gross et al., 1997; Blanchard-Fields & Coats, 2008; Blanke et al., 2020), indicating that depressive symptoms of empty-nest older adults may be associated with the infrequent use of protective passive strategies.

Nevertheless, the present study found that empty-nest older adults with high depressive symptoms used more suppression and less acceptance. This may relate to the adaptive versus maladaptive nature of these strategies: suppression is maladaptive, whereas acceptance is adaptive (Aldao et al., 2010; Aldao & Nolen-Hoeksema, 2010); frequent use of suppression rather than acceptance can exacerbate depressive symptoms (Nezlek & Kuppens, 2008; Kökönyei et al., 2024; Joormann & Stanton, 2016). Thus, depressive symptoms in this population appear to be associated with a pattern of “more maladaptive suppression, less adaptive acceptance.”

Notably, the study also observed that empty-nest older adults with high depressive symptoms used avoidance/denial less frequently in sadness. In contrast, Daros et al. (2021) describe avoidance as a maladaptive strategy whose greater use correlates with emotional problems. This discrepancy may stem from insufficient sensitivity of the measures used here or subtype conceptualization issues. Future work should better distinguish adaptive from maladaptive passive subtypes to clarify their connections to depression.

### 4.2. Proactive and Problem-Solving Strategies in Sad Contexts

In sadness-related interpersonal contexts, empty-nest older adults with high depressive symptoms used significantly fewer Express and Solve strategies, along with related strategies—such as communication, advice-seeking, planning, and problem-solving—compared to their peers with low depressive symptoms. These findings align with the hypothesis and are also consistent with previous studies indicating that depression is often accompanied by interpersonal features such as emotional withdrawal, reduced initiative, and social disengagement (Dawood et al., 2013; Thompson et al., 2018).

Depressed individuals typically show a diminished need for and pleasure in social contact, and tend to avoid emotional expression (Blanchard et al., 2001; Visted et al., 2018). Since proactive social engagement and emotional disclosure are central to interpersonal emotional exchange, their reluctance to initiate such interactions, coupled with decreased social interest, may limit opportunities to maintain existing relationships and form new ones, potentially exacerbating isolation and narrowing their social networks (Liu et al., 2024). Consistent with this view, our sample also showed that empty-nest older adults with high depressive symptoms had significantly lower interaction frequencies with relatives and friends compared to their counterparts with low depressive symptoms.

Thus, in interpersonal sadness contexts, depressive symptoms among empty-nest older adults appear to be associated with less frequent use of adaptive interpersonal-interaction strategies—such as initiating conversations and offering solutions—a pattern that may further reinforce social isolation. The specific reasons remain to be confirmed through further causal research.

### 4.3. Proactive and Problem-Solving Strategies in Anger Contexts

In anger-related contexts, empty-nest older adults with high depressive symptoms used Express and Seek strategies—along with their subtypes, such as expressing feelings and understanding feelings—significantly more frequently than their peers with low depressive symptoms, which is consistent with the research hypothesis. Both Express and Seek are proactive strategies. However, “expressing feelings” and “understanding feelings” tend to reflect covert, internally focused regulation rather than overt, interpersonal strategies (e.g., communication, seeking advice, and obtaining emotional support). This suggests that empty-nest older adults with high depressive symptoms are more inclined to adopt covert intrapersonal regulatory strategies to handle interpersonal anger than their peers with low depressive symptoms.

This pattern aligns with previous findings on covert hostility and retaliatory tendencies in depressed individuals (Dawood et al., 2013; Simon et al., 2015; Cain et al., 2012; Aspland et al., 2008) and may be driven by low self-efficacy and fear of escalating interpersonal conflict (Barrett & Barber, 2007; Constantino et al., 2008). Existing research indicates that depressed individuals generally express emotions less often (Liu et al., 2024), but this reduction may primarily concern emotions such as sadness. In contrast, they may be more prone to expressing anger in anger-eliciting contexts. It is important to note that expressing anger—particularly under high emotional arousal—can itself impair interpersonal relationships (Coats & Blanchard-Fields, 2008).

Therefore, depressive symptoms among empty-nest older adults may be associated with their greater use of covert intrapersonal regulatory strategies in anger contexts. Such strategies not only fail to alleviate negative emotions effectively but may also further worsen their interpersonal relationships. The causal relationships among these variables also need further investigation.

### 4.4. Regulatory Variability Across Interpersonal Contexts

Empty-nest older adults with high depressive symptoms varied their use of only two strategy subtypes across sadness and anger contexts, in contrast to older adults with low depressive symptoms, who varied their use of all seven proactive and problem-solving subtypes. This aligns with evidence linking greater emotion regulation variability to lower negative emotion (Blanke et al., 2020; Wang et al., 2021). The variability observed in empty-nest older adults with low depressive symptoms reflects adaptive flexibility in strategy use (Aldao et al., 2015) and is supported by a wider strategy repertoire (Bonanno & Burton, 2013). Thus, depressive symptoms in this population may relate to lower variability and a narrower regulatory repertoire. Given the cross-sectional design, causality remains unclear, and longitudinal studies are needed to test whether variability predicts symptom onset or whether symptoms reduce flexibility.

## 5. Limitations

This study has several limitations. First, as a cross-sectional design, it cannot establish causality between emotion regulation patterns and depressive symptoms, only their correlation. Longitudinal studies are needed to verify causal pathways. Second, reliance on single-scenario questionnaires may be influenced by subjective interpretation, limiting ecological validity. Future work could adopt multi-scenario designs (e.g., 3–4 typical interpersonal situations per target emotion) to enhance situational coverage and realism. Third, the extreme group comparison used here (high vs. low scorers) may overestimate effects and reduce generalizability. Future studies should analyze depressive symptoms as a continuous variable using the full sample to improve scientific rigor and external validity. Finally, the study’s dependence on a simple questionnaire may restrict the depth of insights. Future research could strengthen reliability and applicability by incorporating mixed methods—such as in-depth interviews, behavioral experiments, or longitudinal tracking—particularly focusing on the elderly population group.

## 6. Future Directions

Despite these limitations, the findings offer useful insights. Contrary to the association often reported between avoidance and emotional problems (Daros et al., 2021), this study did not find that older adults with high depressive symptoms used avoidance more frequently in sadness contexts. Future work should further distinguish adaptive from maladaptive subtypes of passive strategies, clarify concepts like avoidance/denial, and examine their connections to depression. Furthermore, exploring contexts beyond sadness and anger—including positive (e.g., joy) and threat-related (e.g., fear) situations—could help clarify a wider spectrum of regulation patterns linked to depressive symptoms in this group.

## 7. Conclusions

The present study suggests that depressive symptoms among empty-nest older adults are associated with maladaptive emotion regulation patterns in interpersonal contexts. Specifically, these patterns are characterized by a reduced use of passive strategies (e.g., acceptance, avoidance), increased suppression coupled with less proactive interpersonal engagement (e.g., communication, problem-solving) in sadness, more inward-directed aggression in anger, and lower cross-situational variability in strategy use. In China, where family- (often non-co-resident children) and community-based eldercare jointly shape older adults’ support systems, community services and caregivers may use these findings as an initial reference to promote social connectedness and adaptive interpersonal regulation. Further longitudinal and large-sample studies are required before broad practical or clinical implementation.

## Figures and Tables

**Table 1:** Emotion regulation strategies in sadness and anger between empty-nest older adults with high and low depressive symptoms.

Strategy Category	Empty-Nest Older Adults with Low Depressive Symptoms (*n* = 74)		Empty-Nest Older Adults with High Depressive Symptoms (*n* = 55)		*t*	*p*
	*M*	*SD*	*M*	*SD*		
Sadness						
Passive strategies	20.09	3.72	18.35	4.12	2.52 *	0.013
Proactive: Express	15.00	3.15	13.4	3.30	2.80 *	0.006
Proactive: Seek	13.09	3.78	11.78	3.92	1.92	0.057
Solve	13.08	2.37	10.25	3.63	5.34 **	0.000
Anger						
Passive strategies	19.76	3.72	16.64	4.74	4.19 **	0.000
Proactive: Express	8.85	3.81	10.82	4.50	−2.68 *	0.008
Proactive: Seek	9.24	3.19	10.85	4.63	−2.34 *	0.021
Solve	9.22	3.48	9.89	3.88	−1.04	0.302

Note. ** *p* < 0.001, * *p* < 0.05.

**Table 2:** Emotion regulation subtypes in sadness and anger between empty-nest older adults with high and low depressive symptoms.

Strategy Category	Subtypes	Empty-Nest Older Adults with Low Depressive Symptoms (*M* ± *SD*)	Empty-Nest Older Adults with High Depressive Symptoms (*M* ± *SD*)	*t*	*p*
sadness					
Passive	Avoidance/denial	8.43 ± 2.25	7.13 ± 2.21	3.28 **	0.001
	Acceptance	6.89 ± 1.29	5.71 ± 1.64	4.59 **	0.000
	Suppression	4.77 ± 2.14	5.51 ± 1.89	−2.03 *	0.044
Express	Express feelings	9.35 ± 2.53	9.27 ± 2.42	0.18	0.859
	Communication	5.65 ± 1.59	4.13 ± 1.69	5.23 **	0.000
Seek	Understand feelings	4.14 ± 1.41	4.24 ± 1.57	−0.38	0.702
	Get advice	4.32 ± 2.10	3.45 ± 1.78	2.48 *	0.015
	Emotional support	4.64 ± 1.84	4.09 ± 1.78	1.69	0.094
Solve	Problem solve	6.76 ± 1.47	5.15 ± 1.97	5.33 **	0.000
	Plan	6.32 ± 1.21	5.11 ± 1.93	4.39 **	0.000
Anger					
Passive	Avoidance/denial	8.53 ± 1.69	7.13 ± 2.18	4.11 **	0.000
	Acceptance	6.70 ± 1.58	5.25 ± 1.98	4.62 **	0.000
	Suppression	4.53 ± 2.21	4.25 ± 2.27	0.69	0.495
Express	Express feelings	5.51 ± 2.97	7.11 ± 3.44	−2.82 *	0.006
	Communication	3.34 ± 1.40	3.71 ± 1.70	−1.36	0.176
Seek	Understand feelings	2.95 ± 1.27	4.05 ± 2.03	−3.80 **	0.000
	Get advice	2.68 ± 1.31	3.05 ± 1.82	−1.37	0.172
	Emotional support	3.62 ± 1.66	3.75 ± 1.99	−0.38	0.701
Solve	Problem solve	4.70 ± 2.11	5.09 ± 2.23	−1.01	0.315
	Plan	4.51 ± 1.84	4.80 ± 2.16	−0.812	0.419

Note. ** *p* < 0.001, * *p* < 0.05.

**Table 3:** Within-group differences in emotion regulation across sadness and anger between empty-nest older adults with high and low depressive symptoms.

Group	Strategy Category	Subtypes	Sadness (*M* ± *SD*)	Anger (*M* ± *SD*)	*t*	*p*
Empty-nest older adults with low depressive symptoms (*n* = 74)	Passive	Avoidance/denial	8.43 ± 2.25	8.53 ± 1.69	−0.35	0.73
		Acceptance	6.89 ± 1.29	6.70 ± 1.58	0.91	0.37
		Suppression	4.77 ± 2.14	4.53 ± 2.21	0.93	0.36
	Express	Express feelings	9.35 ± 2.53	5.51 ± 2.97	8.99 **	0.000
		Communication	5.65 ± 1.59	3.34 ± 1.40	9.44 **	0.000
	Seek	Understand feelings	4.14 ± 1.41	2.95 ± 1.27	5.13 **	0.000
		Get advice	4.32 ± 2.10	2.68 ± 1.31	6.11 **	0.000
		Emotional support	4.64 ± 1.84	3.62 ± 1.66	4.53 **	0.000
	Solve	Problem solve	6.76 ± 1.47	4.70 ± 2.11	6.65 **	0.000
		Plan	6.32 ± 1.21	4.51 ± 1.84	6.83 **	0.000
Empty-nest older adults with high depressive symptoms (*n* = 55)	Passive	Avoidance/denial	7.13 ± 2.21	7.13 ± 2.18	0.00	1.000
		Acceptance	5.71 ± 1.64	5.25 ± 1.98	1.88	0.066
		Suppression	5.51 ± 1.89	4.25 ± 2.27	3.76 **	0.000
	Express	Express feelings	9.27 ± 2.42	7.11 ± 3.44	4.28 **	0.000
		Communication	4.13 ± 1.69	3.71 ± 1.70	1.52	0.135
	Seek	Understand feelings	4.24 ± 1.57	4.05 ± 2.03	0.69	0.493
		Get advice	3.45 ± 1.78	3.05 ± 1.82	1.51	0.138
		Emotional support	4.09 ± 1.78	3.75 ± 1.99	1.37	0.176
	Solve	Problem solve	5.15 ± 1.97	5.09 ± 2.23	0.14	0.893
		Plan	5.11 ± 1.93	4.80 ± 2.16	0.88	0.382

Note. ** *p* < 0.001.

## Data Availability

The data and analysis scripts are available at the Open Science Framework Repository https://osf.io/678ug (accessed on 6 September 2025).

## Supplementary Materials

The following supporting information can be downloaded at: https://www.mdpi.com/article/10.3390/bs16020263/s1. Questionnaire S1: The Centre for Epidemiological Studies Depression Scale (CES-D10); Questionnaire S2: Strategy Questionnaire (22 items); Questionnaire S3: The Community Screening Interview for Dementia (CSI-D); Questionnaire S4: The 10-item Geriatric Anxiety Scale–Short Form (GAS-10); Questionnaire S5: The Barthel Index (BI); Questionnaire S6: Lawton Instrumental Activities of Daily Living Scale (Lawton-IADL).
