# Supplementary material for: Maladaptive Emotion Regulation Among Empty-Nest Older Adults with Depressive Symptoms Across Interpersonal Contexts
Source: Behav Sci (Basel). 2026 Feb 11;16(2):263. doi: 10.3390/bs16020263 (PMC12938166; doi:10.3390/bs16020263)
Supplement: Supplementary file 1 [file behavsci-16-00263-s001.zip › behavsci-4076597-supplementary.pdf]

Supplementary materials for

Maladaptive Emotion Regulation Among Empty-Nest Older Adults with Depressive Symptoms Across Interpersonal Contexts

Questionnaire used in the study

S1. the Centre for Epidemiological Studies Depression Scale (CES-D10)

Please rate according to your mood over the past week by checking the appropriate option. 0 = rarely or not at all, 1 = not much, 2 = sometimes or about half the time, 3 = most of the time.

1. I was bothered by things that don't usually don't bother me.

2. I had trouble keeping my mind on what I was doing.

3. I felt depressed.

4. I felt that everything I did was an effort.

*5. I felt hopeful about the future.

6. I felt fearful.

7. My sleep was restless.

*8. I felt happy.

9. I felt lonely.

10. I could not get "going".

S2. Strategy Questionnaire (22 items)

Imagine that while you are experiencing this problem, you feel angry (sad). How likely would you be to do each of the following things to handle your ANGER (SADNESS)? Please indicate your response on a 4-point scale (1=*I wouldn't do this at all*, 4=*I would definitely do this*.)

1. I would ask people who have had similar experiences what they did. (SEEK; GET ADVICE)

2. I would concentrate my efforts on doing something about the problem. (SOLVE; PROBLEM SOLVE)

3. I would turn to substitute activities to take my mind off things. (PASSIVE; AVOIDANCE/DENIAL)

4. I would learn to live with it. (PASSIVE; ACCEPT PROBLEM)

5. I would pretend that the problem hasn't really happened. (PASSIVE; AVOIDANCE/DENIAL)

6. I would try to get emotional support from friends or relatives. (SEEK; EMOTIONAL SUPPORT)

7. I would accept that this has happened and that it can't be changed. (PASSIVE; ACCEPT PROBLEM)

8. I would refuse to believe that the problem has happened. (PASSIVE; AVOIDANCE/DENIAL)

9. I would talk to someone about how I feel. (SEEK; EMOTIONAL SUPPORT)

10. I would delve into my feelings to get a thorough understanding of them. (SEEK; UNDERSTAND FEELINGS)

11. I would try to hide my feelings. (PASSIVE; SUPPRESSION)

12. I would try to get advice from someone about what to do. (SEEK; GET ADVICE)

13. I would make a plan of action to try to solve the problem. (SOLVE; PLAN)

14. I wouldn't let anyone see how I'm really feeling. (PASSIVE; SUPPRESSION)

15. I would take action to try to get rid of the problem. (SOLVE; PROBLEM SOLVE)

16. I would try to come up with a strategy about what to do to solve the problem. (SOLVE; PLAN)

17. I would let my feelings come out freely. (EXPRESS; EXPRESS FEELINGS)

18. I would allow myself to express my emotions. (EXPRESS; EXPRESS FEELINGS)

19. I would feel free to express my emotions. (EXPRESS; EXPRESS FEELINGS)

20. I would take time to figure out what I'm really feeling. (SEEK; UNDERSTAND FEELINGS)

21. I would talk to my friend about the problem. (EXPRESS; COMMUNICATION)

22. I would confront my friend about the problem. (EXPRESS; COMMUNICATION)

S3. **The Community Screening Interview for Dementia (CSI-D)**

This questionnaire includes 7 items: Each correct answer is scored as 1 point. For item 7, recalling 1 word scores 1 point, 2 words score 2 points, and 3 words score 3 points. The maximum total score is 9. A total score of ≤7 is considered abnormal.

1. Naming the elbow

2. Stating the use of a hammer

3. Identifying the nearest market/shop

4. Identifying the day of the week

5. Identifying the current season

6. Pointing from the window to the door

7. Delayed recall of 3 words.

S4.The 10-item Geriatric Anxiety Scale–Short Form (GAS-10)

Please answer according to your actual feelings. 0 =not at all, 1 = sometimes, 2 =often, 3 =always. 0-6 points → indicates no anxiety, no need for concern.

1. I was irritable
2. I felt detached or isolated from others
3. I felt like I was in a daze
4. I had a hard time sitting still
5. I could not control my worry
6. I felt restless, keyed up, or on edge
7. I felt tired
8. My muscles were tense
9. I felt like I had no control over my life
10. I felt like something terrible was going to happen to me

S5. The Barthel Index (BI)

This questionnaire is used to assess a patient's ability to perform activities of daily living. It consists of 10 items covering 10 aspects of daily life, including:

① Bowel control,

② Bladder control,

③ Grooming,

④ Toileting,

⑤ Feeding,

⑥ Bathing,

⑦ Dressing,

⑧ Transfers,

⑨ Mobility

⑩ Stair climbing.

Among these, items 1, 2, 4, 5, 7, and 10 are scored from 0 to 2 points; items 8 and 9 are scored from 0 to 3 points; and items 3 and 6 are scored from 0 to 1 point. The total score is 20, with higher scores indicating better ability in activities of daily living.

S6. Lawton Instrumental Activities of Daily Living Scale (Lawton-IADL)

This scale includes 8 items. Each item is worth 1 point if performed independently. The maximum score is 5 for men and 8 for women. A higher score indicates greater independence in daily living activities and relatively better quality of life among older adults.

1. Ability to use telephone
2. Shopping
3. Food Preparation (for women)
4. Housekeeping (for women)
5. Laundry (for women)
6. Mode of Transporation
7. Responsibility for own Medications
8. Ability to Handle Finances
